# Prevalence and Clinical Associations of Antiphospholipid Antibodies in Systemic Sclerosis: New Data From a French Cross-Sectional Study, Systematic Review, and Meta-Analysis

**DOI:** 10.3389/fimmu.2018.02457

**Published:** 2018-11-02

**Authors:** Vincent Sobanski, Angélique Lemaire-Olivier, Jonathan Giovannelli, Luc Dauchet, Myriam Simon, Benjamin Lopez, Cécile Yelnik, Marc Lambert, Pierre-Yves Hatron, Eric Hachulla, Sylvain Dubucquoi, David Launay

**Affiliations:** ^1^Univ. Lille, U995, Lille Inflammation Research International Center, Lille, France; ^2^Inserm, Lille, France; ^3^CHU Lille, Département de Médecine Interne et Immunologie Clinique, Lille, France; ^4^Centre National de Référence Maladies Systémiques et Auto-Immunes Rares (Sclérodermie Systémique), Lille, France; ^5^Inserm UMR1167, RID-AGE, Risk Factors and Molecular Determinants of Aging-Related Diseases, Université de Lille, Centre Hosp. Univ. Lille, Institut Pasteur de Lille, Lille, France; ^6^CHU Lille, Institut d'Immunologie, Lille, France

**Keywords:** systemic sclerosis, antiphospholipid antibodies, pulmonary hypertension, venous thrombosis, miscarriage

## Abstract

**Objectives:** Antiphospholipid antibodies (aPL) can be present in the sera of systemic sclerosis (SSc) patients. This study aimed to determine the prevalence of aPL in a cross-sectional study of SSc patients, to assess their clinical associations, to perform a systematic review of published reports and a meta-analysis to estimate the worldwide prevalence of aPL in SSc.

**Methods:** Two-hundred and forty-nine SSc patients were consecutively tested once for lupus anticoagulant (LA), anticardiolipin (aCL), and anti-β2glycoprotein I (anti-β2GpI) antibodies. Clinical associations with aPL positivity were studied using a logistic regression model. A systematic review of the literature was carried out in PubMed and Embase. Meta-analysis was performed using number of aPL positive (at least one of the three antibodies positive) and negative patients. Meta-regression was used to study potential factors explaining the heterogeneity between studies.

**Results:** In our cross-sectional study, aPL positivity was found in 16 patients (prevalence 6.4%; 95%CI [3.8–10.4]). In multivariate analysis, there was a significant association between aPL positivity and venous thrombosis (VT) (OR 6.25 [1.18–33.00]; *p* = 0.028) and miscarriage (OR 5.43; 95%CI [1.31–22.13]; *p* = 0.017). Twenty-four studies were included in the meta-analysis, representing a total population of 3036 SSc patients. The overall pooled prevalence of aPL in SSc was 14% (9–20) with a high degree of heterogeneity among studies.

**Conclusion:** This study found a prevalence of aPL positivity in our SSc population of 6.4% (3.8–10.4) and an overall worldwide pooled prevalence of 14% (9–20). In our SSc population, aPL positivity was associated with VT and miscarriage. These data provide additional insights into the role of aPL in the vasculopathy observed in SSc.

## Introduction

Systemic sclerosis (SSc) is a severe and chronic connective tissue disorder with skin and internal organ involvement. Immune activation, vasculopathy, and excessive synthesis of extracellular matrix with collagen deposition are known to play a role in the pathophysiology of this disease (1). In SSc, vasculopathy can manifest by Raynaud's phenomenon, digital ulcers (DU), pulmonary arterial hypertension (PAH) as well as venous thrombosis (VT) (1, 2). Many autoantibodies can be detected in patients' sera. The most common are antinuclear auto-antibodies as anti-centromere (ACA), anti-topoisomerase I (anti-topo I), and anti-RNA polymerase III (anti-RNA pol III) antibodies (3). There are some evidences that certain SSc specific autoantibodies, but also newly discovered endothelium-related antibodies, are associated to vasculopathy (4). For example, an association between levels of antibodies and vascular manifestations has been described for antibodies against angiotensin II type 1 receptor and endothelin-1 type A receptor (5). Among antibodies with a possible association with vasculopathy in SSc, antiphospholipid antibodies (aPL) are a heterogeneous group.

The aPL, namely lupus anticoagulant (LA), anticardiolipin antibody (aCL), or anti-β2 glycoprotein-I antibody (anti-β2GpI) are usually found in the primary antiphospholipid syndrome (APS), but can be associated with other connective tissue diseases (mainly systemic lupus erythematosus), infections, drugs, and malignancies. In connective tissue diseases, the significance of aPL in patients who have never suffered from a thrombotic event remains unclear, but could reflect the endothelial activation (6).

In the literature, there are important variations (from 0 to 57%) in the prevalence of aPL in SSc. Moreover, associations of these antibodies with thrombotic events, miscarriage, or SSc clinical manifestations are still debated (7). Some studies reported an association between aPL positivity in SSc and PAH (8–10), digital ulceration (DU) (10, 11), interstitial lung disease (ILD) (10), while others did not (12, 13). Most of these studies have tested a relatively small number of patients, which could be responsible for a lower statistical power. These heterogeneous results preclude any firm conclusion on a link between aPL positivity and clinical manifestation in SSc.

The aims of this study were: (i) to determine the prevalence of aPL in a new cross-sectional study of well-phenotyped SSc patients (ii) to assess their clinical associations with a focus on vasculopathy (iii) to perform a systematic review and a meta-analysis of published reports to estimate the worldwide prevalence of aPL in SSc and to assess the factors associated with the observed heterogeneity.

## Patients and methods

### Patients included in this study

#### Population

Two hundred and forty-nine unselected patients with SSc were consecutively included and studied in the Internal Medicine Department of University Hospital of Lille, France, between October 2014 and January 2016. Patients fulfilled the following criteria for inclusion: age>18 years, and a diagnosis of SSc according to ACR/EULAR criteria (14). Disease subtype was classified based on LeRoy and Medsger criteria: diffuse cutaneous SSc (dcSSc) and limited cutaneous SSc (lcSSc) (15).

#### Data collection

All variables were entered into a standardized questionnaire fulfilled by the clinician at the time of the inclusion. In all patients, at the time of inclusion, the complete medical history of the patients was retrospectively reviewed. Physical examination variables, laboratory and imaging exams were prospectively collected for all patients. Systemic hypertension was defined as blood pressure ≥140/90 mmHg after 10 min of rest. Interstitial lung disease (ILD) was defined as subpleural pure ground-glass opacities and/or interstitial reticular pattern with or without traction bronchiectasis and/or honeycomb cysts, on high resolution computed tomography (HRCT). PAH was diagnosed based on right-heart catheterization if mean pulmonary arterial pressure was ≥25 mmHg and pulmonary capillary wedge pressure ≤ 15 mmHg in a patient with either no ILD or ILD with forced vital capacity % predicted ≥70% and extent of ILD on HRCT ≤ 20% (16). Scleroderma renal crisis (SRC) was defined as the abrupt onset of severe hypertension and/or decline in renal function, with proteinuria without an alternate etiology. The 2001 European and Scleroderma Trials And Research (EUSTAR) disease activity score was calculated for each patient as described in (17).

#### Biological parameters

All patients were tested for LA, aCL (IgG isotype) and anti-β2GpI (IgG isotype). LA was detected in plasma by a dilute Russell's viper venom time (Siemens), and partial thromboplastin time test (HemosIL Silica Clotting Time Werfen) as screening and confirmation tests with calculating a normalized ratio. aCL and anti-β2GpI were measured using commercial ELISA assays (Orgentec, Trappes, France), positive titer was defined as ≥10 UGPL/mL (aCL) or ≥10 UA /mL (anti-β2GpI). Identification of antinuclear antibody specificities using both specific immunofluorescence patterns on HEp-2 cells and the Luminex approach (Bio-Plex 2200; Bio-Rad) for anti–topo I, ACA, anti–U1 RNP, anti-SSA/Ro, and anti-SSB/La antibodies was performed as part of routine clinical care. Anti–RNA pol III antibodies were identified by immunodot (Euroline Systemic Sclerosis [Nucleoli] Profile [IgG]; Euroimmun). Other laboratories tests performed at the inclusion were: creatinine, CRP, platelet count, uric acid, serum protein electrophoresis, immunoglobulin G, M, and A plasma levels, LDL-cholesterol, triglycerides and glycated hemoglobin. Diabetes mellitus was defined as a glycated hemoglobin ≥6.5% and/or anti-diabetic medication intake. Dyslipidemia was defined as a LDL-cholesterol ≥1.6 g/L and/or triglycerides ≥1.5 g/L and/or lipid-lowering medication intake.

#### Ethics approval and consent to participate

This study was authorized by the French Competent Authority dealing with Research on Human Biological Samples namely the French Ministry of Research. The Authorization number is DC 2008 642. To issue such authorization, the Ministry of Research has sought the advice of an independent ethics committee, namely the “Comité de Protection des Personnes,” which voted positively. French legislation on non-interventional studies requires collecting the non-opposition of patients but does not require written consent. As such, non-opposition was obtained from each patient included in the study for the use of their de-identified medical record data.

### Systematic review and meta-analysis

The statement on Preferred Reporting Items for Systematic Reviews and Meta-Analyses (PRISMA) was used as a guide to conduct the review and analysis (18).

#### Search strategy

Two of the authors (VS and AL) performed a search of published studies between May 1975 and November 2015, in PubMed and Embase databases. We used combinations of the terms “systemic sclerosis,” “scleroderma,” “antibodies, antiphospholipid,” “antibody syndrome, antiphospholipid,” “lupus anticoagulant,” “antibodies, anticardiolipin,” “antibodies, anti-β2GP1,” “thrombosis,” “pulmonary embolism,” “digital ulceration,” “pulmonary hypertension,” “deep vein thrombosis,” “cavernous sinus thrombosis,” “stroke,” “myocardial infarction,” “acute limb ischemia,” “pregnancy,” “miscarriage.” We adapted the search strategy to meet the specificities of each database. The reference lists of the retrieved reports were searched to identify additional relevant publications.

#### Study selection

Inclusion criteria were: French or English-language publication, patients >18 years old, and diagnosed as having SSc, and at least 30 patients with SSc were tested in each study for LA, or/and aCL or/and anti-β2GpI. Reports that failed to provide sufficient information for the data analysis were excluded. Two of the authors (VS and AL) independently screened the titles and abstracts of the articles that were retrieved and applied the selection criteria to identify relevant material to be read in full. The reviewers' selections were compared and, in cases of disagreement, a third author (DL) was involved and decisions were made by consensus. The reviewers independently read the complete articles and applied the selection criteria to determine whether the studies would be included in the meta-analysis. The selections were again compared, and in cases of disagreement, a third author (DL) was again involved and decisions were made by consensus. Since the studies that were initially selected included some overlapping cohorts for a given center assessed during the same period, we chose to include 1 study per center (whichever study included the highest number of patients). Studies of 2 or more cohorts were included if extraction of data for each cohort was feasible. In this case, each cohort was analyzed as an independent cohort. Multicenter studies were excluded if participating centers had published single-cohort reports that were already included. Therefore, for each center, only 1 source of information was analyzed in order to avoid duplicate data (19).

#### Quality

Two authors (VS and AL) independently assessed the quality of the studies (risk of bias) using the Quality Assessment of Diagnostic Accuracy Studies (QUADAS-2) tool (16). In accordance with the QUADAS-2 user guidelines (16), items were modified for this study. In domain 1 (Patient selection), the item “Was a case–control design avoided?” was omitted. In domain 2 (Index test), the items “Were the index test results interpreted without knowledge of the results of the reference standard?” and “If a threshold was used, was it pre-specified?” were substituted with the item “Was the method of antibody determination described?” In domain 3 (Reference standard), the items “Is the reference standard likely to correctly classify the target condition?” and “Were the reference standard results interpreted without knowledge of the results of the index test?” were omitted. In domain 4 (Flow and timing), the item “Was there an appropriate interval between index test and reference standard?” was omitted, and the item “Did all patients receive the same reference standard?” was substituted with the item “Were all patients tested for aPL?” In accordance with the QUADAS-2 guidelines, articles were assessed for each item according to the following rating scale: high risk of bias, low risk of bias, or unclear (Supplementary Table 8).

#### Data extraction

Following information were extracted from each selected study: continent, country, center, disease duration, disease subtype (percentage of diffuse form), age of patients, sex ratio, percentage of patients with ILD, DU, SRC, PAH, ACA, and anti-topo I, number of patients tested for aPL, number of patients positive for aPL and which type of aPL (LA, aCL, or anti-β2GpI) and isotype of aCL and anti-β2GpI (IgG and/or IgM). Thrombosis and miscarriage events were not collected because of missing data or high variability in definitions. Authors were contacted in case of missing data for the calculation of aPL prevalence.

### Statistical analysis

Characteristics of patients were described using mean and standard deviation (SD) for continuous variables and count and percentage for categorical variables. Characteristics of patients as a function of aPL status (aPL+/aPL–) were compared using Student's test for continuous variables and Fisher's exact test for categorical variables. The associations between aPL status and complications (arterial or venous thrombosis, miscarriage, PAH, and DU) were studied using binomial logistic regressions. Adjustments were done (i) a priori for gender, age at aPL testing, SSc type (dc/lcSSc) and disease duration, and (ii) for the characteristics that differed significantly between aPL+ and aPL– patients (*p* < 0.20). Regression diagnostics were performed.

**Figure 1:**
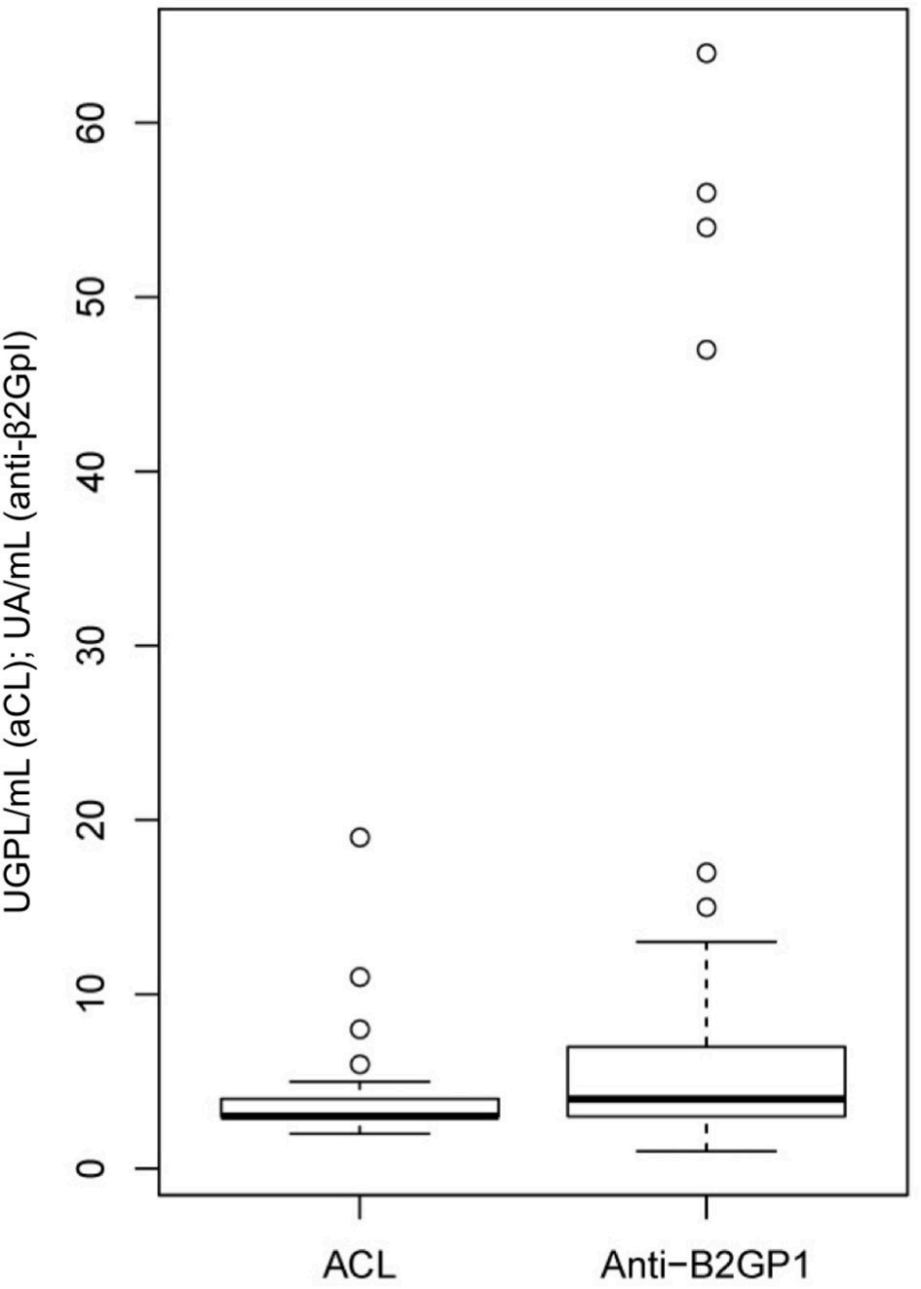
Titers of aCL and anti-β2GpI in this cross-sectional study of SSc patients.

Similar analyzes were performed considering the titers of aCL and anti-β2GpI rather than the aPL status. Because these variables had a majority of zero values, they were categorized (visual analysis of their distribution as shown in Figure 1 and Supplementary Figure 1), as follows: (i) 0, ≥1 and < 5, ≥5 and ≤ 20 UGPL/mL for aCL, and (ii) 0, ≥1 and < 5, ≥5 and < 10, ≥10 and ≤ 100 UA/mL for anti-β2GpI.

As a sensitivity analysis, we then focused on patients who were tested twice for LA, aCL, and anti-β2GpI (“repeat testing”). Patients with “persistent aPL” were defined as having the same positive test for at least one aPL at two different times. We performed similar comparisons and associations studies than with the “single testing.”

For the meta-analysis, we calculated weighted pooled summary estimates of aPL prevalence. For each meta-analysis, the DerSimonian-Laird method was used. Accordingly, studies were considered to be a random sample from a population of studies. Heterogeneity was quantified using a chi-square heterogeneity statistic and by means of an *I*^2^ statistic for each analysis. A random-effects model was used to combine data. The overall effect was estimated using a weighted average of the individual effects, with weights inversely proportional to variance in observed effects. Freeman-Tukey transformation was used. Meta-regression was performed to assess the impact of continent, country, center, disease duration, disease subtype (percentage of diffuse form), age of patients, sex ratio, percentage of patients with ILD, DU, SRC, PAH, ACA, and anti-topo I, risk of bias using QUADAS-2 tool. For aCL and anti-β2GpI prevalence, the isotype of aCL and anti-β2GpI tested (IgG and/or IgM) were also included, respectively.

All statistical analyses were performed using R software, version 3.1.2 (20); the threshold for statistical significance was set to *p* < 0.05. For meta-analysis, R metafor package was used.

## Results

### Lille cross-sectional study

#### Patients and disease characteristics

The 249 patients included in our study were predominantly female (82%), with lcSSc (82%). Mean age of patients at the time of the study was 59.5 ± 13.3 years and mean disease duration was 10.7 ± 8.9 years (Table 1). The prevalence of ILD was 45%, DU 33% and PAH 6%. Forty-five (18%) patients had a history of arterial (*n* = 22) or venous (*n* = 29) thrombosis, and 40 (21%) patients had a history of miscarriage (characteristics of the individual thrombotic events and miscarriage are described in Supplementary Table 1).

**Table 1:** Characteristics of the population included in the study, and comparison between aPL positive or negative patients (single testing).

	**N (N aPL+)**	**Whole population (*n* = 249)**	**aPL+(*n* = 16)**	**aPL–(*n* = 233)**	***p***
Sex, *n* (%) female	249 (16)	205 (82)	14 (88)	191 (82)	0.745
Age, mean ± SD years	249 (16)	59.5 ± 13.3	65.9 ± 7.4	59.1 ± 13.5	**0.047**
Age at onset of disease, mean ± SD years	204 (11)	47.7 ± 13.7	48.2 ± 11.5	47.6 ± 13.9	0.897
Disease duration, mean ± SD years	204 (11)	10.7 ± 8.9	15.3 ± 10.9	10.5 ± 8.8	0.082
BMI mean ± SD	232 (16)	25.1 ± 5.7	29.2 ± 8.3	24.8 ± 5.4	**0.003**
Tobacco use, n (%)	248 (16)	99 (40)	2 (13)	97 (42)	**0.032**
Systemic hypertension, n (%)	249 (16)	125 (50)	11 (69)	114 (49)	0.195
Diabetes mellitus, n (%)	249 (16)	12 (5)	1 (6)	11 (5)	0.558
Dyslipidemia, n (%)	249 (16)	112 (45)	11 (69)	101 (43)	0.068
Disease subtype n (%)	249 (16)				
Limited		203 (82)	15 (94)	188 (81)	0.318
Diffuse		46 (18)	1 (6)	45 (19)	
mRSS, mean ± SD	247 (16)	5.1 ± 5.8	4.3 ± 4.5	5.1 ± 5.9	0.581
Pulmonary arterial hypertension, *n* (%)	233 (16)	15 (6)	1 (6)	14 (6)	1.000
Interstitial lung disease, *n* (%)	230 (16)	104 (45)	6 (38)	98 (46)	0.608
Digital ulceration, *n* (%)	236 (14)	79 (33)	2 (14)	77 (35)	0.150
Renal crisis, *n* (%)	231 (16)	1 (0)	0	1 (1)	1.000
2001 EUSTAR SSc activity score	234 (16)	1.2 ± 1.2	1.2 ± 1.1	1.2 ± 1.2	0.943
Arterial or venous thrombosis, *n* (%)	246 (16)	45 (18)	6 (38)	39 (17)	0.086
Arterial thrombosis, *n* (%)	247 (16)	22 (9)	3 (19)	19 (8)	0.160
Stroke/transient ischemic attack		11 (4)	2 (13)	9 (4)	0.154
Acute limb ischemia		3 (1)	0	3 (1)	1.000
Myocardial infarction		5 (2)	1 (6)	4 (2)	0.287
Venous thrombosis, n (%)	248 (16)	29 (12)	5 (31)	24 (10)	**0.027**
DVT		22 (9)	4 (25)	18 (8)	**0.041**
PE		9 (4)	2 (13)	7 (3)	0.108
Miscarriage, *n* (%)	187 (12)	40 (21)	5 (42)	35 (20)	0.136
ANA specificity, *n* (%)	238 (16)				
ACA		139 (58)	12 (75)	127 (57)	0.196
Anti–topo I		50 (21)	3 (19)	47 (21)	1.000
Anti-RNA pol III		7 (3)	0	7 (3)	1.000
Anti–U1RNP		9 (4)	0	9 (4)	1.000
Patients ANA negative, *n* (%)		2 (1)	0	2 (1)	1.000
CRP > 10 mg/L, *n* (%)	248 (15)	21 (8)	1 (7)	20 (9)	1.000
Hypergammaglobulinemia, *n* (%)	249 (16)	30 (12)	1 (6)	29 (12)	0.701
HbA1c > 6.5%, *n* (%)	247 (16)	6 (2)	0	6 (3)	1.000

*N, number of patients (whole population) with available data; N aPL+, number of patients aPL+ with available data; ANA, antinuclear antibody; anti–topo I, anti–topoisomerase I antibody; ACA, anticentromere antibody; Anti-RNA pol III, anti-RNA polymerase III antibody; Anti-U1RNP, Anti-U1RNP antibody; CRP, C-reactive protein; DVT, Deep venous thrombosis; PE, pulmonary embolism; mRSS, mean Rodnan skin score. Values in bold are significant p < 0.05*.

#### Frequency of aPL

One or more aPL were found in 16 (6.4%, 95%CI [3.8–10.4]) patients. One patient was positive for both LA and anti-β2GpI, and one was positive for aCL and anti-β2GpI. The prevalence of positive LA was 1.6% (0.4–4.1). The prevalence of aCL was 1.2% (0.3–3.5) with a mean value of 14 UGPL/mL for positive patients. The prevalence of anti-β2GpI was 4.4% (2.3–8.0) with a mean value of 28 UA/mL for positive patients (Figure 1).

Among patients with aPL, LA was found in 25.0%, aCL in 18.8%, and anti-β2GpI in 68.8% (Table 2). Double positivity was seen in 12.5%. At the time of the study, 2 patients had been previously diagnosed with an APS based on clinical and biological criteria. Among the 29 patients with a history of VT, five had aPL positivity (LA and/or anti-β2GpI, but no aCL), 2 were diagnosed as having APS (they were both positive for LA, none of them were positive for anti-DNA antibodies or fulfilled systemic lupus erythematosus criteria).

**Table 2:** Prevalence of aPL in this cross-sectional SSc study and frequencies of LA, aCL, and anti-β2GpI in SSc patients with aPL (*n* = 249, single testing).

	**Prevalence of aPL in this study (% and 95% CI)**	**Frequencies of LA, aCL and anti-β2GpI in SSc patients with aPL (%)**
≥ 1 aPL	6.4 (3.8–10.4)	–
LA	1.6 (0.4–4.1)	25.0
aCL	1.2 (0.3–3.5)	18.8
Anti-β2GpI	4.4 (2.3–8.0)	68.8

#### Associations with clinical manifestations

The associations of aPL positivity with disease manifestations are presented in Table 1 and Supplementary Table 2. Mean age of aPL positive patients was higher than aPL negative patient (65.9 ± 7.4 vs. 59.1 ± 13.5 yrs, *p* = 0.047). A higher BMI (29.2 ± 8.3 vs. 24.8 ± 5.4, *p* = 0.003) was found in aPL positive patients group, while tobacco use was less frequent (13% vs. 42%, *p* = 0.032). No difference was found regarding disease subtype, ILD, DU, autoantibodies status, CRP elevation, HbA1c > 6.5%, as well as hypergammaglobulinemia.

In univariate analysis, aPL positivity was associated with an increased risk of VT (OR = 3.91; 95%CI [0.98–13.53]; *p* = 0.027). No association was found between aPL positivity and arterial thrombosis, miscarriage, PAH, ILD, DU, and renal crisis. When adjusted on sex, age at aPL testing, disease duration at aPL testing, disease subtype, tobacco use, BMI, systemic hypertension, dyslipidemia and ACA positivity, aPL positivity was significantly associated with VT (OR = 6.25 [1.18–33.00]; *p* = 0.028) and miscarriage (OR = 5.43; [1.31–22.13]; *p* = 0.017) (Table 3).

**Table 3:** Univariate and multivariate comparisons of associations between aPL positivity in SSc patients and clinical manifestations.

	**Univariate OR (95% CI)**	***p***	Multivariate OR (95% CI) ^*^	***p***
Arterial or venous thrombosis	2.92 (0.82–9.51)	0.086	**5.21 (1.18–23.20)**	**0.027**
Arterial thrombosis	2.56 (0.43–10.55)	0.160	2.50 (0.31–13.90)	0.323
Venous thrombosis	**3.91 (0.98–13.53)**	**0.027**	**6.25 (1.18–33.00)**	**0.028**
Miscarriage	2.84 (0.67–11.11)	0.136	**5.43 (1.31–22.13)**	**0.017**
Digital ulceration	0.32 (0.03–1.47)	0.150	0.48 (0.07–2.29)	0.400
Pulmonary arterial hypertension	0.97 (0.02–7.26)	1	0.72 (0.03–6.37)	0.790

* *OR adjusted for sex, age at aPL testing, disease duration at aPL testing, disease subtype, tobacco use, BMI, systemic hypertension, dyslipidemia, ACA positivity. Values in bold are significant p < 0.05*.

We then focused on aPL titers and their clinical associations. Distribution of aCL and anti-β2GpI are shown in Figure 1 and Supplementary Figure 1. In multivariate analysis (model adjusted on sex, age, disease subtype, tobacco use, follow-up, gammaglobulin level, and anti-U1RNP positivity), there was an association between aCL titers (≥5 UGPL/mL) and VT (OR 3.69; [0.98–12.9]; *p* = 0.043) as well as with PAH (OR 6.35; [0.99–41.1]; *p* = 0.043). Anti-β2GpI titer ≥10 UA/mL was associated with an increased risk of miscarriage (OR 5.19; [0.99–28.4]; *p* = 0.049) in multivariate analysis (model adjusted on sex, age, disease subtype, tobacco use, dyslipidemia, BMI, gammaglobulin level, ACA positivity, and anti-topo I positivity) (Table 4, Supplementary Tables 3, 4).

**Table 4:** Multivariate comparisons of associations between aCL and anti-β2GpI titers and clinical manifestations.

	**aCL titers (UGPL/mL)**^**a**^					**Anti-**β**2GpI titers (UA/mL)**^**b**^						
	**[0,1[**	**[1,5[**		**[5,20]**		**[0,1[**	**[1,5[**		**[5,10[**		**[10,100]**	
	**Ref**	**Adjusted OR (CI)**	***p***	**Adjusted OR (CI)**	***p***	**Ref**	**Adjusted OR (CI)**	***p***	**Adjusted OR (CI)**	***p***	**Adjusted OR (CI)**	***p***
Arterial or venous thrombosis		1.12 (0.50–2.44)	0.780	2.66 (0.83–8.10)	0.088		0.55 (0.21–1.37)	0.211	0.62 (0.16–1.96)	0.443	3.27 (0.77–13.6	0.101
Arterial thrombosis		0.84 (0.28–2.34)	0.739	1.52 (0.30–6.17)	0.576		0.54 (0.13–1.90)	0.356	0.58 (0.08–2.54)	0.511	3.76 (0.61–2.08)	0.131
Venous thrombosis		1.71 (0.67–4.36)	0.257	**3.69 (0.98–12.9)**	**0.043**		0.79 (0.26–2.29)	0.669	0.55 (0.08–2.33)	0.466	3.44 (0.72–15.3)	0.106
Miscarriage		1.43 (0.64–3.19)	0.381	0.62 (0.09–2.62)	0.557		1.04 (0.41–2.58)	0.933	0.56 (0.11–2.04)	0.413	**5.19 (0.99–28.4)**	**0.049**
Digital ulceration		0.66 (0.33–1.28)	0.226	1.85 (0.64–5.39)	0.253		0.47 (0.21–1.02)	0.06	0.78 (0.31–1.85)^*^		0.573^*^	
PAH		3.65 (0.94–17.9)	0.074	**6.35 (0.99–41.1)**	**0.043**		0.98 (0.27–3.42)	0.977	0.25 (0.01–1.76)^*^		0.237^*^	

a *Adjusted on sex, age, disease subtype, tobacco use, follow-up, gammaglobulin level, anti-U1RNP positivity*.

b *Adjusted on sex, age, disease subtype, tobacco use, dysplidemia, BMI, gammaglobulin level, ACA positivity, anti-topo I positivity; Ref: class reference*;

* *class: [5,100]. Values in bold are significant p < 0.05*.

As a sensitivity analysis, we then focused on patients who were tested twice for LA, aCL, and anti-β2GpI (repeat testing, *n* = 213). The time interval between the two assessments of aPL were 13.2 ± 6.3 months. Seven patients were found with persistent aPL corresponding to a prevalence of 3.3% (1.5–6.9). The prevalence of patients with persistent LA, aCL, and anti-β2GpI were 0.9% (0.1–3.4), 0 and 2.4% (0.9–5.7), respectively. Patients with persistent aPL had higher BMI (33.3 ± 8.6 vs. 24.9 ± 5.4, *p* < 0.001), and higher rates of VT (57 vs. 11%, *p* = 0.006) and miscarriage (67 vs. 21%, *p* = 0.023) than patients with aPL negative or non-persistent aPL. In univariate and multivariate analysis, persistent aPL were associated with venous thrombosis (multivariate OR 7.93 [1.38–53.40]; *p* = 0.022) and miscarriage (multivariate OR 18.35 [2.83–163.34]; *p* = 0.003) (Supplementary Tables 5–7).

### Systematic review and meta-analysis

#### Studies included

**Figure 2:**
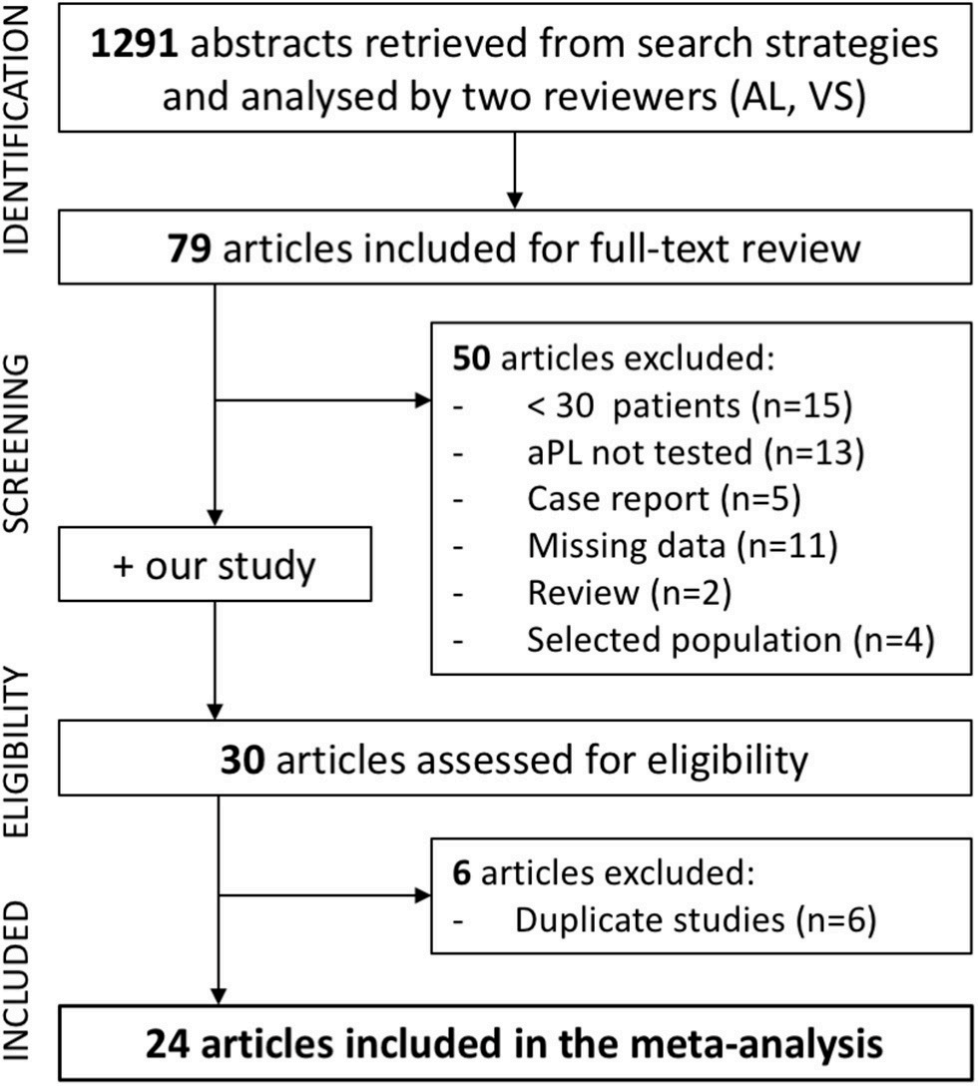
Flowchart illustrating the study selection process for meta-analysis of the prevalence of aPL in SSc.

One thousand and two hundred and ninety-one references were retrieved as result of search (575 articles in Pubmed and 716 in Embase). Seventy-nine articles were included for full text review after reading the titles and abstracts. Of these articles, 30 were assessed for eligibility. Six articles were further excluded (duplicate studies) (Figure 2). Finally, 24 studies (23 + our study) were included in the meta-analysis, representing a total population of 3,036 adult patients with SSc (8–10, 12, 13, 21–38) (Supplementary Table 8). One study (26) provided the prevalence of LA, aCL, and anti-β2GpI but did not provide the global prevalence of aPL. This study was therefore included in the meta-analysis excepted for the calculation of the global prevalence.

#### Prevalence of APL

**Figure 3:**
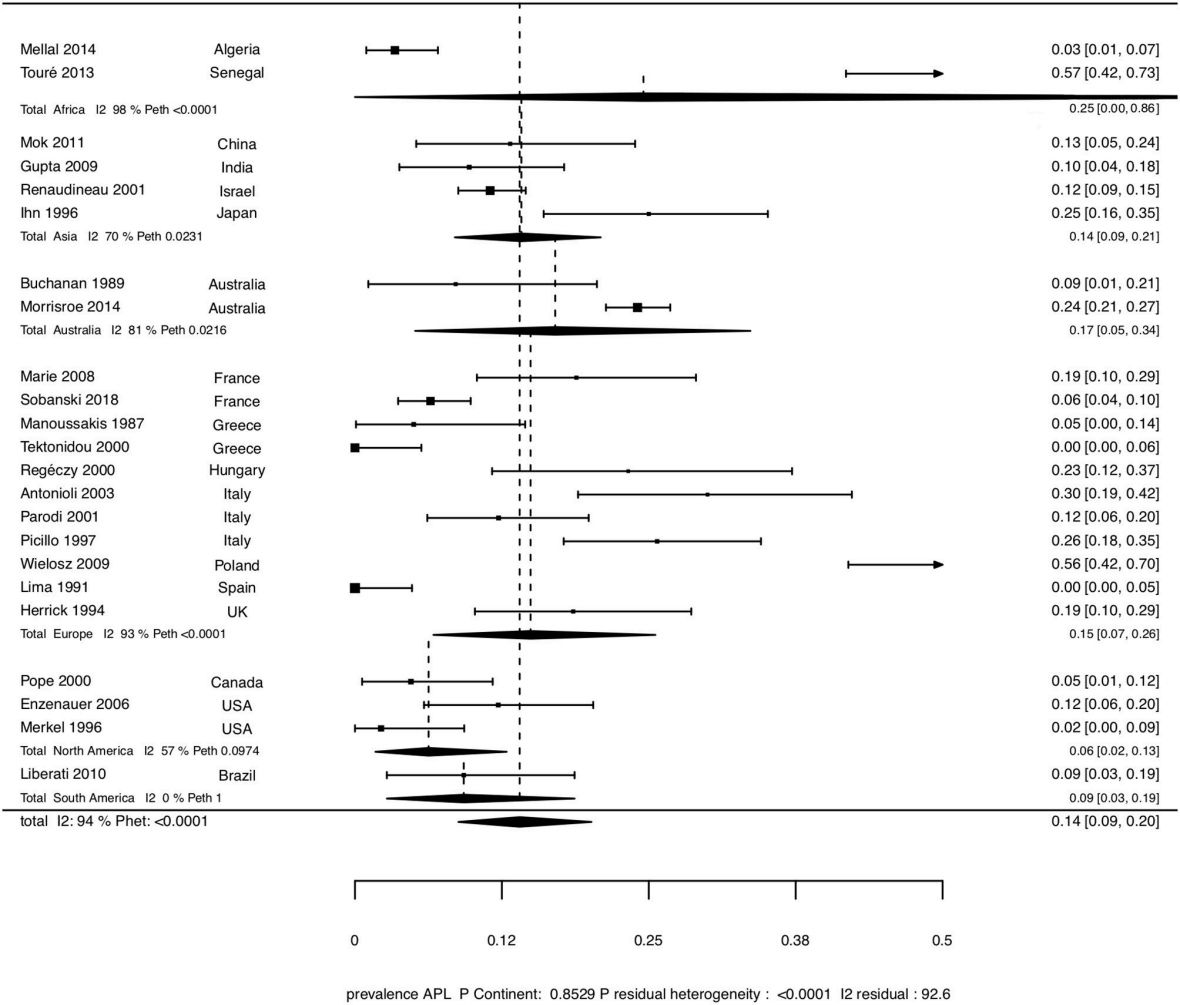
Forest plot showing the pooled prevalence of aPL in the whole population of SSc patients, and stratified by continent.

The overall pooled prevalence of aPL was 14% (95%CI [9-20]), with a high degree of heterogeneity (*I*^2^ = 94%, *P* < 0.0001). The overall pooled prevalence of LA, aCL, and anti-β2GpI were 1% (0–3), 9% (6–13), and 9% (3–18), respectively. There was a high degree of heterogeneity (*I*^2^ = 81%, *P* < 0.0001 for LA, *I*^2^ = 87%, *P* < 0.0001, for aCL, and *I*^2^ = 95%, *P* < 0.0001 for anti-β2GpI). Pooled prevalence stratified by continent are shown in Table 5 and Figure 3.

**Table 5:** Prevalence of aPL, LA, aCL, and anti-β2GpI in SSc, stratified by continent.

	**aPL (%[95%CI]) *N* = 23**	**LA (%[95%CI]) *N* = 6**	**aCL (%[95%CI]) *N* = 21**	**anti-β2GpI (%[95%CI]) *N* = 9**
Africa	25 [0–86]	5 [0–15]	9 [0–27]	50 [35–65]
	*N* = 2	*N* = 1	*N* = 2	*N* = 1
Asia	14 [9–21]	3 [0–8]	14 [6-24]	10 [4–18]
	*N* = 4	*N* = 1	*N* = 3	*N* = 1
Oceania	17 [5–34]	0 [0–0]	10 [8–12]	6 [5–8]
	*N* = 2	*N* = 1	*N* = 2	*N* = 1
Europe	15 [7–26]	1 [0–3]	9 [4–16]	5 [1–11]
	*N* = 11	*N* = 3	*N* = 10	*N* = 6
North America	6 [2–13]	NA	6 [2–13]	NA
	*N* = 3		*N* = 3	
South America	9 [3–19]	NA	9 [3–19]	NA
	*N* = 1		*N* = 1	

*N, number of studies with available data; NA, not available*.

We assessed whether the characteristics of the studies included could explain the observed heterogeneity. Table 6 summarizes the results of all meta-regression analyses. Meta-regression revealed a significant association between the sex ratio and the prevalence of aPL positivity (*p* = 0.0265). However, when sex ratio was entered as a variable in the model, residual heterogeneity between studies remained significant (*P* < 0.0001, *I*^2^ = 91.8%). Meta-regression did not find any other factors significantly associated with aPL prevalence: continent, disease subtype, disease duration, age, proportion of patients with ILD, DU, SRC, PAH, ACA, and anti-topo I. Meta-regression revealed a significant association between prevalence of LA and disease duration (*p* < 0.0001), between prevalence of aCL and proportion of patients with ACA (*p* = 0.0055), and between prevalence of anti-β2GpI and continent (*p* = 0.0040), age (*p* = 0.0333), sex ratio (*p* = 0.0469), respectively. Yet, excepted for disease duration and prevalence of LA (residual heterogeneity *p* = 0.428, *I*^2^ = 0%), residual heterogeneity was still significant (*P* < 0.0001) after inclusion of these factors in the models. The tested isotype of aCL and anti-β2GpI were not associated with the prevalence of their respective antibodies (Table 6).

**Table 6:** Results of meta-regression analyses of the associations between aPL prevalence and characteristics of the studies included.

**Variable**	**P value for association of variable with prevalence**			
	**aPL**	**LA**	**aCL**	**anti-β2GpI**
Continent	0.8529	0.1964	0.9387	**0.0040**
Disease subtype	0.5226	0.9850	0.1734	0.7101
Disease duration	0.8790	<**0.0001**	0.7068	0.5799
Age	0.7507	0.1755	0.4863	**0.0333**
Gender	**0.0265**	0.1690	0.9489	**0.0469**
ILD	0.2986	0.4585	0.8599	0.2105
DU	0.5581	0.4516	0.3294	0.7851
SRC	0.0809	0.2796	0.6226	0.9635
PAH	0.4690	0.3098	0.2763	0.8342
ACA	0.1795	0.5451	**0.0055**	0.8613
Anti-topo I	0.3705	0.7974	0.2068	0.6560
Tested isotype of aCL	—	—	0.3580	—
Tested isotype of anti-β2GpI	—	—	—	0.2847
Risk of bias (QUADAS-2)	0.1510	0.9881	0.2766	0.8717

*Values in bold are significant p < 0.05*.

**Figure 4:**
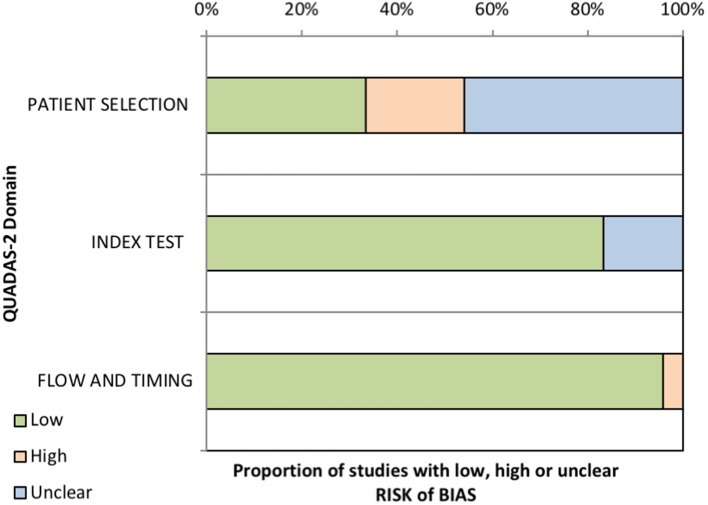
Results of the methodologic assessment using the QUADAS-2 tool showing the proportion of studies with low, high, or unclear risk of bias.

Since quality bias is an important concern in meta-analyses, we assessed the quality of the studies using the QUADAS-2 tool (Figure 4 and Supplementary Table 8). Meta-regression did not find any association between the risk of bias and the prevalence of aPL (Table 6). Moreover, the aforementioned analyses were performed in duplicate for the five studies for which both LA, aCL and anti-β2GpI were tested [(8, 10, 22, 26), in this study]. The prevalence of aPL, LA, aCL, and anti-β2GpI were 24% (7–47), 1% (0–4), 11% (4–19), and 10% (1–27), respectively. There was still a high degree of heterogeneity (*I*^2^ = 98, 84, 93, and 97% for aPL, LA, aCL, and anti-β2GpI prevalence respectively, *P* < 0.0001).

## Discussion

The main results of our study were as follows: (1) the prevalence of aPL in this population of SSc patients was 6.4% (1.6, 1.2, 4.4% for LA, aCL, and anti-β2GpI, respectively) and the overall pooled prevalence of aPL in SSc was 14% (9–20) by meta-analysis, (2) there was an association between VT and miscarriage and aPL positivity, (3) higher levels of aCL were associated with a higher risk of PAH and VT, and higher levels of anti-β2GpI with a higher risk of miscarriage.

The prevalence of aPL in SSc in our study was 6.4% (3.8–10.4), which is rather low compared to previous reports. Although the prevalence of aPL ranges from 0 to 57% in the literature (8, 10, 12, 13, 21–27, 29–40), most of the studies reported an overall prevalence higher than 10% and the overall pooled prevalence of aPL in SSc was 14% (9–20) as found by our meta-analysis. The important variation in the prevalence of aPL reported in literature was associated with a high heterogeneity among studies included in our meta-analysis. The first hypothesis to explain this heterogeneity was the influence of methodological differences: different positivity cutoff, different type of aPL tested and subgroups analysis. One of the most important factors which may induce variations of prevalence of aPL was the aPL isotypes tested. Indeed, some studies only analyzed IgG-aPL isotype, other analyzed IgG and IgM-aPL isotype, and even some analyzed IgG, IgM, and IgA isotypes of aPL. IgG is the most prevalent isotype among patients with thrombosis and fetal loss in APS, and the only one associated with these events (39). Our meta-regression analysis did not find any association between isotype and aCL and anti-β2GpI prevalence. Disease duration was associated with LA prevalence, but this should be interpreted with caution given the small number of studies included (5) and the low prevalence of LA (between 0 and 4% in these studies). The observed heterogeneity could also be explained by the geographic origin of the studies, because of potential genetic or environmental factors. Indeed, Touré et al. reported the highest prevalence in the review of literature (57%), with an African ethnicity cohort (22) while studies from North and South America found a prevalence range of 2.2–12.2% (13, 36–38). However, meta regression did not show an association between continent and aPL positivity (*p* = 0.8529), but there was an association between continent and anti-β2GpI positivity (*P* = 0.0040). It has been reported that aPL positivity and APS were associated with various HLA alleles (41). Interestingly, gender was associated with aPL and anti-β2GpI prevalence. Age of patients was also associated with anti-β2GpI prevalence. Yet, these results explained only very partially the marked heterogeneity among studies.

We found an association between aPL positivity and VT in univariate and in multivariate analysis. This association is not common in studies of SSc patients and most of them did not find an increased risk of VT in case of aPL positivity (8, 10, 12, 13, 22). Yet, Antonioli et al. reported an association between thrombosis (arterial or venous) and aPL positivity (30). Interestingly, SSc has been recently reported to be associated with a higher risk of VT when compared to non-SSc individuals with a HR of 2.96 (1.54–5.69), particularly in the first year following the diagnosis of SSc (2). There are a number of possible mechanisms that could account for the increased risk of VTE seen in SSc patients, in particular vasculopathy and low-grade inflammation. Moreover, obesity is a common risk factor for VT. In our study, patients aPL positive had a higher BMI than those who were aPL negative. BMI, tobacco use and disease duration were included in our multivariate analysis.

Our study also showed an increased risk of miscarriage in case of aPL positivity, and this association was significant in multivariate analysis. One of our patients with miscarriage history was known to have an APS. Mean age at onset of the disease was 47.7 years, meaning that most patients had already finished childbearing at that time. In our study, SSc had been diagnosed more than 2 years after the last miscarriage in 25/40 women who had experienced miscarriage and with available data. Interestingly, we also reported a significant association between higher titers of anti-β2GpI and risk of miscarriage. To our knowledge, there is no previously published data on these findings in SSc. This result might be linked with the pathogenic role of aPL in fetal loss observed in APS. Indeed, aPL (in particular β2GpI-dependant antibodies) bind to human trophoblasts and affect several cell function *in vitro* (42).

Using the manufacturer cut-off levels, we did not show any association between aPL positivity and other clinical manifestations, in particular with PAH and DU. This may be due to a lack of power (small number of events). Regarding the association between aPL positivity and PAH, results in literature are discrepant. Marie et al. reported an increased risk of PAH in case of one or more aPL positivity (8). Antonioli et al., Assous et al., and Morrisroe et al. reported an association between aCL positivity and PAH (10, 26, 30), while Boin et al. found this association with anti-β2GpI (11) in a selected population. On the other hand, Gupta et al., Enzenauer et al., did not report this association. Touré et al. found a trend (12, 13, 22). As in our study, studies that did not highlight an association between PAH and aPL positivity had a low prevalence of aPL (9.1–14%) (12, 13). Moreover, we defined PAH by hemodynamics during a right heart catheterization, which is the gold standard and not by echocardiography. We cannot rule out to have overlooked PAH in some patients but, in the same way, some patients diagnosed as having PAH only on echocardiography and not hemodynamics in other studies could also have been misclassified.

Interestingly, considering the titers of aPL rather than the aPL status positivity/negativity, we found that higher titers of aCL ≥5 UGPL/mL were associated with PAH. This is consistent with the existing literature. Morrisroe et al. identified that higher titers of aCL-IgG corresponded with a higher risk of PAH in SSc patients (10). Assous et al. identified a trend toward an association between a higher mean titer of aCL and PAH (*p* = 0.06). They also found an association between patients with PAH and the amount of von Willebrand factor produced (26). It has been shown that endothelial cell injury in SSc patients was accompanied by an elevation in the level of von Willebrand factor (43). This suggests that aCL positivity could be associated with endothelial injury and PAH in SSc. It has also been reported that there was an increased amount of E-selectin in patients with aPL positivity (with or without APS), and an increased amount of P-selectin and sVCAM-1 in patient with APS (44, 45). These three molecules are also involved in pathogenesis of SSc and PAH (1, 46).

Our study has several limitations. First, we chose to quantify only IgG subtype of aCL and anti-β2GpI, because IgG was the most prevalent isotype among patient with thrombosis and fetal loss in APS, and the only one associated with these events (39). However, an overall screening of anti-β2GpI IgG, IgM, and IgA subtype was done. IgM and IgA were often more prevalent in the studies in which they are quantified (10, 11). Secondly, due to the low prevalence of aPL, there might be a lack of power. Moreover, some clinical events were quite rare in aPL positive patients (PAH in one patient, miscarriage in 5) appealing for a cautious interpretation of these data. Thirdly thrombosis history and miscarriage has been collected retrospectively, leading to a potential memorization bias and a selection bias (in classification of APS patients). It is difficult to avoid this bias, because obstetrical events occurred mainly years before the onset of SSc. A prospective study would be needed to avoid these biases.

In conclusion, this study found a prevalence of aPL in SSc of 6.4% (3.8–10.4) and an overall pooled prevalence of 14% (9–20). aPL positivity was associated with VT and miscarriage. These data provide additional insights into the role of aPL in the vasculopathy observed in SSc.

## Author contributions

VS, AL-O, SD, and DL: design of study; VS, AL-O, MS, BL, CY, ML, P-YH, EH, SD, and DL: collection of data; JG and LD: statistical analysis; VS, AL-O, JG, LD, SD, and DL: redaction of manuscript; VS, AL-O, JG, LD, MS, BL, CY, ML, P-YH, EH, SD, and DL: critical review of manuscript.

### Conflict of interest statement

The authors declare that the research was conducted in the absence of any commercial or financial relationships that could be construed as a potential conflict of interest. The handling Editor declared a past co-authorship in the last 2 years with one of the authors, DL.
