# Supplementary material for: Prevalence and Clinical Associations of Antiphospholipid Antibodies in Systemic Sclerosis: New Data From a French Cross-Sectional Study, Systematic Review, and Meta-Analysis
Source: Front Immunol. 2018 Nov 2;9:2457. doi: 10.3389/fimmu.2018.02457 (PMC6234954; doi:10.3389/fimmu.2018.02457)
Supplement: Supplementary file 1 [file Data_Sheet_1.docx]

**Supplementary material**

**Supplementary table 1.** Characteristics of SSc patients included in this study who had a thrombotic event.

| **Patient**  **and sex** | | **SSc subtype** | **Thrombotic event** | **Disease duration at the time of the event (years)** | **LA** | **aCL (UGPL/mL)** | **Anti -β2GP1 (UA/mL)** | **Time between event and aPL testing (years)** | **Age at the event (years)** | **APS criteria** |
| --- | --- | --- | --- | --- | --- | --- | --- | --- | --- | --- |
| 6 | F | lcSSc | PE | +9 | neg | 0 | 0 | 3 | 71 |  |
| 8 | F | lcSSc | ALI | 0 | neg | 0 | 0 | 4 | 52 |  |
| **9** | **F** | **lcSSc** | **PE** | **+7** | **pos** | **5** | **56** | **9** | **50** | **Yes** |
| 18 | F | lcSSc | DVT1  DVT2 | - 37  - 33 | neg | 3 | 4 | 40  36 | 22  26 |  |
| 38 | M | lcSSc | Stroke/TIA | + 8 | neg | 0 | 0 | 1 | 68 |  |
| 48 | F | lcSSc | DVT | 0 | neg | 0 | 0 | 27 | 53 |  |
| 52 | F | lcSSc | DVT  MI | - 5  + 8 | neg | 4 | 4 | 27  3 | 62  75 |  |
| 60 | M | lcSSc | BTUA | 0 | neg | 0 | 5 | 1 | 59 |  |
| 62 | F | lcSSc | PE  BTUA | +1  0 | neg | 0 | 0 | 2  3 | 41  40 |  |
| 65 | M | lcSSc | ALI | NA | neg | 4 | 7 | NA | NA |  |
| 72 | F | lcSSc | DVT | NA | neg | 0 | 0 | NA | NA |  |
| 85 | F | lcSSc | 3 DVT | NA | neg | 4 | 3 | NA | NA |  |
| 89 | M | lcSSc | 3 DVT | NA | neg | 3 | 0 | NA | NA |  |
| 96 | F | lcSSc | DVT | NA | neg | 0 | 0 | NA | NA |  |
| **97** | **F** | **lcSSc** | **DVT1**  **DVT2**  **DVT 3**  **Stroke/TIA** | **+ 5**  **+ 6**  **+ 7**  **- 3** | **neg** | **5** | **47** | **2**  **1**  **0**  **10** | **65**  **66**  **67**  **57** |  |
| **101** | **F** | **lcSSc** | **DVT** | **0** | **pos** | **0** | **4** | **17** | **54** | **Yes** |
| 105 | F | dcSSc | MI, Stroke/TIA | + 7 | na | 0 | 0 | 1 | 73 |  |
| 107 | F | lcSSc | DVT | - 12 | neg | 3 | 3 | 41 | 28 |  |
| **112** | **F** | lcSSc | **DVT1**  **4 DVT, 1 PE**  **MI** | **+ 18**  **NA**  **+ 19** | **neg** | **4** | **15** | **3**  **NA**  **2** | **65**  **NA**  **66** |  |
| 121 | F | lcSSc | DVT | + 20 | neg | 4 | 6 | 11 | 35 |  |
| 123 | F | lcSSc | DVT 1  DVT 2 | + 14  + 28 | neg | 5 | 4 | 15  1 | 58  72 |  |
| 126 | **F** | lcSSc | **Stroke/TIA** | **- 3** | **neg** | **19** | **17** | **8** | **50** |  |
| 130 | F | lcSSc | Stroke/TIA 1  Stroke/TIA 2 | + 7  + 10 | neg | 3 | 0 | 10  7 | 34  37 |  |
| 131 | F | dcSSc | DVT | + 15 | neg | 0 | 0 | 5 | 67 |  |
| 143 | F | lcSSc | DVT | + 3 | neg | 0 | 0 | 4 | 67 |  |
| **145** | **F** | lcSSc | **DVT 1**  **DVT 2** | **- 24**  **+ 3** | **neg** | **4** | **12** | **30**  **3** | **26**  **53** |  |
| 148 | F | lcSSc | Stroke/TIA | - 17 | neg | 5 | 0 | 32 | 36 |  |
| 151 | F | lcSSc | Stroke/TIA | + 15 | neg | 0 | 7 | 8 | 74 |  |
| 156 | F | lcSSc | DVT  Stroke/TIA | + 1  NA | neg | 0 | 0 | 0  NA | 65  NA |  |
| 157 | F | lcSSc | BTUA | + 16 | neg | 3 | 3 | 1 | 68 |  |
| 164 | F | lcSSc | Stroke/TIA | + 8 | neg | 0 | 0 | 1 | 72 |  |
| 170 | F | lcSSc | MI | + 29 | neg | 3 | 4 | 6 | 76 |  |
| 173 | M | lcSSc | BTUA | NA | neg | 3 | 0 | NA | NA |  |
| 176 | F | lcSSc | DVT | + 1 | neg | 3 | 5 | 3 | 31 |  |
| 188 | F | lcSSc | MI | - 19 | neg | 0 | 0 | 25 | 56 |  |
| 191 | F | lcSSc | PE | NA | neg | 0 | 0 | NA | NA |  |
| 192 | M | lcSSc | DVT  ALI | NA  0 | neg | 3 | 3 | NA  18 | NA  39 |  |
| 196 | F | lcSSc | Stroke/TIA | + 16 | neg | 3 | 0 | 1 | 74 |  |
| 197 | F | lcSSc | DVT | - 40 | neg | 0 | 0 | 40 | 37 |  |
| 204 | F | lcSSc | PE | + 1 | neg | 8 | 6 | 18 | 61 |  |
| 207 | F | lcSSc | DVT1, PE 1  DVT2, PE 2 | - 10  + 13 | neg | 5 | 0 | 25  2 | 34  57 |  |
| 215 | F | lcSSc | DVT 1  DVT2  DVT 3 | - 29  - 24  - 4 | neg | 0 | 7 | 34  29  9 | 35  40  60 |  |
| 222 | F | lcSSc | Stroke/TIA | + 29 | neg | 0 | 0 | 1 | 59 |  |
| 227 | F | lcSSc | PE | + 6 | neg | 3 | 0 | 12 | 56 |  |
| 231 | F | dcSSc | PE | + 16 | neg | 3 | 0 | 1 | 39 |  |

lcSSc = limited cutaneous SSc ; dcSSc = diffuse cutaneous SSc ; PE = pulmonary embolism ; DVT = deep venous thrombosis ; MI = myocardial infarction; BTUA = bilateral thrombosis of ulnar arteries; Stroke/TIA = stroke or transient ischemic attack; ALI = acute limb ischemia; pos = positive; neg = negative. APS: antiphospholipid syndrome

Supplementary table 2. Patients’ characteristics according to thrombotic or obstetric history.

|  | Arterial thrombosis | | Venous thrombosis | | Miscarriage | |
| --- | --- | --- | --- | --- | --- | --- |
|  | no | yes | no | yes | no | yes |
| Sex, no. (%) female | 185 (82) | 18 (82) | 178 (81) | 27 (93) | na | na |
| Age, mean (SD) years | **59 (13)**** | **67 (11)**** | **59 (13)*** | **64 (11)*** | 59 (15) | 57 (11) |
| Age at onset of disease, mean (SD) years | 47 (14) | 52 (12) | 47 (14) | 50 (15) | 46 (14) | 46,5 (12) |
| Disease duration, mean (SD) years | 10,4 (8,9) | 12,7 (8,7) | 10,4 (9) | 13,3 (8) | 11,6 (9,2) | 9,8 (7,4) |
| Disease subtype  Limited  Diffuse | 180 (80)ᶿ  45 (20) | 21 (95)ᶿ  1 (5) | 176 (80)  43 (20) | 27 (93)  2 (7) | 129 (88)ᶿ  18 (12)ᶿ | 30 (75)ᶿ  10 (25)ᶿ |
| Pulmonary arterial hypertension, no. (%) | 15 (7) | 0 | 11 (5)ᶿ | 4 (14)ᶿ | 8 (6) | 2 (6) |
| Interstitial lung disease, no. | 93 (45) | 11 (50) | 92 (46) | 11 (41) | 53 (40) | 17 (46) |
| Digital ulceration, no. (%) | 72 (34) | 6 (30) | 71 (34) | 8 (31) | 44 (31) | 15 (38) |
| Renal crisis, no. (%) | 0ᶿ | 1 (5)ᶿ | 1 (1) | 0 | 1 (1) | 0 |
| BMI | 25 (5,7) | 26 (6,3) | 24,9 (5,6) | 26.5 (5,7) | 25,3 (5,3) | 25,2 (7,3) |
| Tobacco | 88 (39) | 9 (41) | 90 (41) | 8 (29) | 52 (35) | 15 (37) |
| ANA specificity, no. (%)  ACA  Anti–topo I  Anti-RNA pol III  Anti–U1RNP | 124 (58)  45 (21)  7 (3)  8 (4) | 13 (59)  5 (23)  0  1 (5) | **117 (56)***  44 (21)  7 (3)  8 (4) | **22 (79)***  5 (18)  0  1 (4) | 92 (66)  29 (21)  4 (3)  7 (5) | 25 (64)  4 (10)  1 (3)  2 (5) |
| CRP (> 10 mg/L) | 19 (8) | 2 (9) | 17 (8) | 4 (14) | 12 (8) | 4 (10) |
| Hypergammaglobulinemia | 29 (13) | 1 (5) | 28 (13) | 2 (7) | 13 (9) | 7 (17) |
| HbA1c (> 6.5%) | 5 (2) | 1 (5) | 3 (1) | 2 (7) | 3 (2) | 1 (3) |
| Rheumatoid factor |  |  |  |  |  |  |
| APL  IgG ACL  IgG anti-B2GpI  LA | 13 (6)  2 (1)  8 (4) ᶿ  4 (2) | 3 (14)  1 (5)  3 (14) ᶿ  0 | **11 (5)***  3 (1)  **7 (3)***  2 (1)ᶿ | **5 (17)***  0  **4 (14)***  2 (7)ᶿ | 7 (5)  2 (1)  4 (3)ᶿ  2 (1) | 5 (12)  0  4 (10)ᶿ  2 (5) |

ᶿ p < 0.10, * p < 0.05, ** p < 0.01, *** p < 0.001

|  | PAH | | UD | |
| --- | --- | --- | --- | --- |
|  | no | yes | no | yes |
| Sex, no. (%) female | 181 (83) | 13 (87) | 131 (83) | 65 (82) |
| Age, mean (SD) years | 59 (13) | 65 (10) | 61 (12)ᶿ | 57 (14)ᶿ |
| Age at onset of disease, mean (SD) years | 48 (14) | 49 (12) | 49 (13)ᶿ | 44 (15) ᶿ |
| Disease duration, mean (SD) years | 10,7 (9) | 13,8 (9,1) | 10,3 (9) | 12,3 (8,9) |
| Disease subtype  Limited  Diffuse | 184 (84)  34 (16) | 10 (67)  5 (33) | **157 (89)***  **18 (11)*** | **55 (70)***  **24 (30)*** |
| Pulmonary arterial hypertension, no. (%) | Na | na | 8 (5) | 7 (9) |
| Interstitial lung disease, no. | 87 (43) | 10 (67) | **53 (37)**** | **40 (56)**** |
| Digital ulceration, no. (%) | 68 (33) | 7 (47) | na | na |
| Renal crisis, no. (%) | 1 (1) | 0 | 1 (1) | 0 |
| BMI | 25 (5,6) | 24,9 (7,6) | **25,7 (5,8)*** | **23,7 (5)*** |
| Tobacco | **91 (42)*** | **2 (13)*** | 62 (39) | 33 (42) |
| ANA specificity, no. (%)  ACA  Anti–topo I  Anti-RNA pol III  Anti–U1RNP | 124 (60)  41 (20)  6 (3)  7 (3) | 7 (47)  5 (33)  0  1 (7) | **98 (65)***  **22 (15)***  4 (3)  8 (5) | **37 (49)***  **22 (29)***  3 (4)  1 (1) |
| CRP (> 10 mg/L) | 20 (9) | 1 (7) | 15 (10) | 5 (6) |
| Hypergammaglobulinemia | 25 (11) | 3 (20) | 15 (10)ᶿ | 14 (18)ᶿ |
| HbA1c (> 6.5%) | 6 (3) | 0 | 5 (3,52) | 1 (1,4) |
| Rheumatoid factor |  |  |  |  |
| APL  IgG ACL  IgG anti-B2GpI  LA | 15 (7)  3 (1)  10 (5)  4 (2) | 1 (7)  0  1 (7)  0 | 12 (8)  1 (1)  **9 (6)***  4 (3) | 2 (3)  2 (3)  **0***  0 |

Supplemental figure 1. Distribution of aCL and anti-B2GpI levels in the study population

Supplementary table 3. Correlation between aCL titers and clinical manifestations

|  | aCL titers (UGPL/mL) | | |  |
| --- | --- | --- | --- | --- |
|  | [0,1[  n=137 | [1,5[  n=91 | [5,20]  n=21 | p |
| Sex, n (%) female | 114 (83) | 73 (80) | 18 (86) | 0.818 |
| Age, mean ± SD years | 58.6 (13.1) | 60.6 (13) | 60.5 (14.9) | 0.504 |
| Age at onset of disease,  mean ± SD years | 48.4 (13.4) | 47.55 (13.9) | 42.69 (14.8) | 0.320 |
| Disease duration, mean ± SD years | 9.19 (7.8) | 12.11 (9.6) | 14.71 (11.2) | 0.016 |
| BMI mean ± SD | 24.5 (5.2) | 25.7 (6.3) | 26.6 (5.6) | 0.132 |
| Tobacco use, n (%) | 57 (42) | 36 (40) | 6 (29) | 0.545 |
| Systemic hypertension, n (%) | 64 (47) | 49 (54) | 12 (57) | 0.443 |
| Diabetes, n (%) | 5 (4) | 7 (8) | 0 | 0.258 |
| Dyslipidemia, n (%) | 61 (45) | 39 (43) | 12 (57) | 0.495 |
| Disease subtype, n (%)  Limited  Diffuse | 113 (82)  24 (18) | 73 (80)  18 (20) | 17 (81)  4 (19) | 0.880  0.880 |
| Pulmonary arterial hypertension, n (%) | 3 (2) | 9 (11) | 3 (16) | 0.007 |
| Interstitial lung disease, n (%) | 61 (49) | 34 (40) | 9 (43) | 0.406 |
| Digital ulceration, n (%) | 46 (35) | 23 (26) | 10 (53) | 0.079 |
| Renal crisis, n (%) | 1 (1) | 0 (0) | 0 (0) | 1 |
| Arterial or venous thrombosis, n (%) | 21 (16) | 17 (19) | 7 (33) | 0.142 |
| Arterial thrombosis, n (%)  Stroke  Ischemia  Myocardial infarction | 11 (8)  6 (4)  1 (1)  2 (1) | 8 (9)  2(2)  2(2)  3(3) | 3 (14)  3(14)  0 (0)  0 (0) | 0.583  0.064  0.668  0.613 |
| Venous thrombosis, n (%)  DVT  PE | 12 (9)  9 (7)  3 (2) | 12 (13)  10 (11)  3 (3) | 5 (24)  3(14)  3 (14) | 0.095  0.280  0.044 |
| Miscarriage, n (%) | 21 (20) | 17 (26) | 2 (13) | 0.521 |
| ANA specificity, n (%)  ACA  Anti–topo I  Anti-RNA pol III  Anti–U1RNP | 55 (42)  27 (21)  5 (4)  1 (1) | 36 (41)  17 (20)  2 (2)  7 (8) | 8 (38)  6 (29)  0 (0)  1 (5) | 0.96  0.619  0.847  0.014 |
| CRP>10 mg/L, n (%) | 11 (8) | 7 (8) | 3 (14) | 0.567 |
| Hypergammaglobulinemia, n (%) | 11 (8) | 17 (19) | 2 (10) | 0.050 |
| HbA1c>6.5%, n (%) | 2 (1) | 4 (4) | 0(0) | 0.341 |

Supplementary table 4. Correlation between anti-β2GpI titers and clinical manifestations

|  | **anti-β2GpI titers (UA/mL)** | | | | |
| --- | --- | --- | --- | --- | --- |
|  | [0,1[  n=120 | [1,5[  n=83 | [5,10[  n=35 | [10,100]  n=11 | p |
| Sex, n (%) female | 100 (83) | 65 (78) | 30 (86) | 10 (91) | 0.683 |
| Age, mean ± SD years | 59.6 (12.7) | 59 (14.5) | 58.8 (13.5) | 65.4 (7.1) | 0.495 |
| Age at onset of disease, mean ± SD years | 47.6 (12.9) | 47.5 (14.7) | 48.1 (15.3) | 48 (8.7) | 0.997 |
| Disease duration, mean ± SD years | 10.9 (9.5) | 10.2 (8) | 10.5 (9) | 13.6 (9.9) | 0.808 |
| BMI mean ± SD | 24.8 (5.6) | 25.1 (5.4) | 25.0 (6.0) | 29.6 (7.2) | 0.059 |
| Tobacco use, n (%) | 50 (42) | 37 (45) | 12 (34) | 0 (0) | 0.019 |
| Systemic hypertension, n (%) | 61 (51) | 41 (49) | 15 (43) | 8 (73) | 0.402 |
| Diabetes, n (%) | 6 (5) | 5 (6) | 1 (3) | 0 | 0.914 |
| Dyslipidemia, n (%) | 59 (49) | 27 (33) | 18 (51) | 8 (73) | 0.017 |
| Disease subtype, n (%)  Limited  Diffuse | 97 (81)  23 (19) | 62 (75)  21 (25) | 33 (94)  2 (6) | 11 (100)  0(0) | 0.027  0.027 |
| Pulmonary arterial hypertension, n (%) | 7 (6) | 7 (9) | 0(0) | 1 (9) | 0.243 |
| Interstitial lung disease, n (%) | 57 (52) | 32 (42) | 12 (36) | 3 (27) | 0.198 |
| Digital ulceration, n (%) | 43 (38) | 23 (29) | 13 (38) | 0 (0) | 0.074 |
| Renal crisis, n (%) | 1 (1) | 0 (0) | 0 (0) | 0 (0) | 1 |
| Arterial or venous thrombosis, n (%) | 24 (20) | 9 (11) | 7(21) | 5 (45) | 0.038 |
| Arterial thrombosis, n (%)  Stroke  Ischemia  Myocardial infarction | 12 (10)  8 (7)  1 (1)  2 (2) | 4 (5)  0 (0)  1 (1)  2 (3) | 3 (9)  1 (3)  1 (3)  0 (0) | 3 (27)  2 (18)  0 (0)  1 (9) | 0.099  0.009  0.603  0.348 |
| Venous thrombosis, n (%)  DVT  PE | 14 (12)  9 (8)  6 (5) | 7 (9)  7 (8)  0 (0) | 4 (11)  3 (9)  1 (3) | 4 (36)  3 (27)  2 (18) | 0.096  0.195  0.016 |
| Miscarriage, n (%) | 19 (21) | 13 (22) | 4 (15) | 4 (50) | 0.237 |
| ANA specificity, n (%)  ACA  Anti–topo I  Anti-RNA pol III  Anti–U1RNP | 61 (54)  19 (17)  5 (4)  4 (4) | 45 (56)  24 (30)  2(3)  5 (6) | 23 (68)  6 (18)  0 (0)  0 (0) | 10 (91)  1 (9)  0 (0)  0 (0) | 0.065  0.117  0.761  0.526 |
| CRP>10 mg/L, n (%) | 10 (8) | 9 (11) | 1 (3) | 1 (9) | 0.534 |
| Hypergammaglobulinemia, n (%) | 13 (11) | 12 (14) | 5 (14) | 0 (0) | 0.593 |
| HbA1c>6.5%, n (%) | 2 (2) | 4 (5) | 0 (0) | 0 (0) | 0.43 |

**Supplementary table 5.** Characteristics of the population included in the study, and comparison between aPL positive (persistent) vs. negative/non-persistent aPL patients (repeat testing).

|  | N (N aPL+) | Whole population  (n=249) | Persistent aPL  (n=7) | aPL negative or non-persistent aPL  (n=206) | p |
| --- | --- | --- | --- | --- | --- |
| Sex, n (%) female | 249 (7) | 205 (82) | 7 (100) | 165 (80) | 0.351 |
| Age, mean ± SD years | 249 (7) | 59.5 ± 13.3 | 64.9 ± 6.4 | 59.0 ± 13.0 | 0.233 |
| Age at onset of disease, mean ± SD years | 204 (6) | 47.7 ± 13.7 | 52.1 ± 6.6 | 47.2 ± 13.6 | 0.381 |
| Disease duration, mean ± SD years | 204 (6) | 10.7 ± 8.9 | 11.6 ± 7.6 | 10.8 ± 8.9 | 0.843 |
| BMI mean ± SD | 232 (7) | 25.1 ± 5.7 | 33.3 ± 8.6 | 24.9 ± 5.4 | **<0.001** |
| Tobacco use, n (%) | 248 (7) | 99 (40) | 1 (14) | 87 (42) | 0.243 |
| Systemic hypertension, n (%) | 249 (7) | 125 (50) | 6 (86) | 99 (48) | 0.063 |
| Diabetes, n (%) | 249 (7) | 12 (5) | 1 (14) | 11 (5) | 0.338 |
| Dyslipidemia, n (%) | 249 (7) | 112 (45) | 5 (71) | 93 (45) | 0.252 |
| Disease subtype n (%)  Limited  Diffuse | 249 (7) | 203 (82)  46 (18) | 7 (100)  0 | 164 (80)  42 (20) | 0.350 |
| Pulmonary arterial hypertension, n (%) | 233 (7) | 15 (6) | 1 (14) | 13 (7) | 0.402 |
| Interstitial lung disease, n (%) | 230 (7) | 104 (45) | 2 (29) | 93 (49) | 0.447 |
| Digital ulceration, n (%) | 236 (6) | 79 (33) | 0 | 71 (36) | 0.092 |
| Renal crisis, n (%) | 231 (7) | 1 (0) | 0 | 1 (1) | 1.000 |
| Arterial or venous thrombosis, n (%) | 246 (7) | 45 (18) | 4 (57) | 33 (16) | 0.019 |
| Arterial thrombosis, n (%)  Stroke/transient ischemic attack  Acute limb ischemia  Myocardial infarction | 247 (7) | 22 (9)  11 (4)  3 (1)  5 (2) | 1 (14)  0  0  1 (14) | 14 (7)  6 (3)  3 (1)  2 (1) | 0.406  1.000  1.000  0.096 |
| Venous thrombosis, n (%)  DVT  PE | 248 (7) | 29 (12)  22 (9)  9 (4) | 4 (57)  3 (43)  2 (29) | 23 (11)  17 (8)  7 (3) | **0.006**  **0.020**  **0.030** |
| Miscarriage, n (%) | 187 (6) | 40 (21) | 4 (67) | 32 (21) | **0.023** |
| ANA specificity, n (%)  ACA  Anti–topo I  Anti-RNA pol III  Anti–U1RNP | 238 (7) | 139 (58)  50 (21)  7 (3)  9 (4) | 6 (86)  1 (14)  0  0 | 109 (55)  45 (23)  4 (2)  7 (4) | 0.140  1.000  1.000  1.000 |
| CRP > 10 mg/L, n (%) | 248 (7) | 21 (8) | 0 | 19 (9) | 1.000 |
| Hypergammaglobulinemia, n (%) | 249 (7) | 30 (12) | 0 | 29 (14) | 0.597 |
| HbA1c > 6.5%, n (%) | 247 (7) | 6 (2) | 0 | 6 (3) | 1.000 |

**Supplementary table 6.** Prevalence of persistent aPL in this study and frequencies of LA, aCL and anti-β2GpI in SSc patients with persistent aPL (n=213, repeat testing).

|  | Prevalence of persistent aPL in this study (% and 95% CI) | Frequencies of LA, aCL and anti-β2GpI in SSc patients with persistent aPL (%) |
| --- | --- | --- |
| ≥ 1 aPL | 3.3 (1.5-6.9) | --- |
| LA | 0.9 (0.1-3.4) | 2 |
| aCL | 0 | 0 |
| Anti-β2GpI | 2.4 (0.9-5.7) | 5 |

**Supplementary table 7.** Univariate and multivariate comparisons of associations between persistent aPL in SSc patients and clinical manifestations.

|  | **Univariate OR (95% CI)** | **p** | **Multivariate OR (95% CI) *** | **p** |
| --- | --- | --- | --- | --- |
| Arterial or venous thrombosis | **6.81 (1.10-48.62)** | **0.019** | **6.65 (1.17-45.10)** | **0.036** |
| Arterial thrombosis | 2.26 (0.05-20.81) | 0.406 | 2.61 (0.12-24.60) | 0.442 |
| Venous thrombosis | **10.33 (1.64-75.04)** | **0.006** | **7.93 (1.38-53.40)** | **0.022** |
| Miscarriage | **7.56 (1.03-87.20)** | **0.023** | **18.35 (2.83-163.34)** | **0.003** |
| Pulmonary arterial hypertension | 2.31 (0.05-21.42) | 0.402 | 1.31 (0.06-10.95) | 0.821 |

* OR adjusted for sex, age at aPL testing, skin involvement, tobacco use, systemic hypertension, ACA positivity

**Supplementary table 8:** Characteristics of the studies included in the meta-analysis and results of methodological quality assessment using the QUADAS-2 tool.

Items of modified QUADAS-2 tool used in this study:

1. Was a consecutive or random sample of patients enrolled?
2. Did the study avoid inappropriate exclusions?
3. Was the method of aPL determination described?
4. Were all patients included in the study tested for aPL?

According to the QUADAS-2 manual, each item was assessed “yes”, “no” or “unclear”:

|  | Yes |
| --- | --- |
|  | No |
|  | Unclear |

| Author, year (ref.) | Country  *Ethnic origin of the patients* | Study design  (time interval) | Length of follow-up or duration of disease | Method of aPL  determination – Cut-off for ELISA | No. of SSc patients positive/tested for aPL | QUADAS | | | |
| --- | --- | --- | --- | --- | --- | --- | --- | --- | --- |
|  |  |  |  |  |  | 1 | 2 | 3 | 4 |
| Antonioli *et al*, 2003 | Italy  *NA* | Cohort (NA) | Mean duration = 13 years (range 1-42) | ELISA (homemade), >99^th^ percentile | 8/60 (aCL-IgG/IgM)  14/60 (anti-β2GP1-IgG/IgM) |  |  |  |  |
| Assous *et al*, 2005 | France  *NA* | Cohort of consecutive patients (2001-2003) | 10 ± 2 yr* | Immunoassays (homemade),  >98^th^ percentile (15 for aCL and 6 for anti-β2GP1) | 0/108 (LA)  15/108 (aCL-IgG/IgM)  5/108 (anti-β2GP1-IgG/IgM) |  |  |  |  |
| Buchanan *et al*, 1989 | Australia  *Caucasian 100%* | NA | NA | ELISA (homemade), mean ± 3 SD of controls (20UI) | 3/35 (aCL-NA) |  |  |  |  |
| Enzenauer *et al*, 2006 | USA  *NA* | NA | NA | ELISA (Biopostar reads), mean ± 2 SD of controls (22.6UI) | 10/82 (aCL-IgG) |  |  |  |  |
| Gupta *et al*, 2009 | India  *NA* | Cross-sectionnal study (2002-2006) | Mean duration = 3 years | ELISA (Genesis Diagnostics), 12UI/mL | 2/72 (LA)  6/72 (aCL-IgG/IgM) |  |  |  |  |
| Herrick *et al*, 1994 | UK  *NA* | 60 unselected patients + 8 with sever ischaemia (NA) | Median duration = 12 years (Raynaud’s onset) | ELISA, 5UI | 13/68 (aCL-IgG) |  |  |  |  |
| Ihn *et al*, 1996 | Japan  *NA* | NA | Mean duration = 7 years | ELISA (homemade), mean ± 3 SD of controls | 20/80 (aCL-IgG)  8/80 (anti-β2GP1-IgG) |  |  |  |  |
| Liberati *et al*, 2010 | Brazil  *NA* | Transversal study (NA) | 12.3 ± 8.9 yr* | ELISA (Euroimmun), 15 UI/mL | 5/54 (aCL-IgG) |  |  |  |  |
| Lima *et al*, 1991 | Spain  *NA* | NA | 10.1 ± 7.7 yr* | ELISA, ≥ 5 UI | 0/35 (aCL-IgG/IgM) |  |  |  |  |
| Manoussakis *et al*, 1987 | Greece  *NA* | Unselected (NA) | 7.0 ± 4.5 yr* | ELISA (homemade), ≥ mean ± 4 SD of controls (2.3 UI) | 2/40 (aCL-IgG/IgM) |  |  |  |  |
| Marie *et al*, 2008 | France  *NA* | Consecutive patients (NA) | Median duration = 5 years (range 1-40) | LA: dilute thromboplastine time  ELISA (INOVA and Hemagen diagnostics), NA | 13/69 (aPL)  3/69 (LA)  13/69 (aCL-IgG/IgM)  3/69 (anti-β2GP1-IgG/IgM) |  |  |  |  |
| Mellal *et al*, 2014 | Algeria  *NA* | NA | 12.2 ± 9.3 yr* | ELISA (INOVA) | 5/147 (aCL-IgG) |  |  |  |  |
| Merkel *et al*, 1996 | USA  *NS* | Inception cohort of newly diagnosed CTD (1982-1987) | NS | ELISA (homemade), ≥ mean ± 5 SD of controls (10 UI) | 1/45 (aCL-IgG) |  |  |  |  |
| Mok *et al*, 2011 | China  *Asian 100%* | Consecutive patients | Median duration = 9 (IQR 5.5-19.5) | LA (cf. Article)  ELISA (homemade), 15 UI | 7/46 (LA+aCL-IgG) |  |  |  |  |
| Morrisroe *et al*, 2014 | Australia  *NA* | Patients from a prospective cohort | 15.3 ± 13.9 yr* | LA (cf. Article)  ELISA (Vital diagnostics for aCL and Orgentec for anti-β2GP1), > 0 UI | 226/940 (LA+aCL-IgG/IgM+ anti-β2GP1-NA)  0/940 (LA)  98/940 (aCL-IgG)  63/940 (anti-β2GP1-NA) |  |  |  |  |
| Parodi *et al*, 2001 | Italy  *NA* | NA | NA | ELISA (Bouty/Orgentec) | 11/90 (aCL-IgG+anti-β2GP1-IgG)  11/90 (aCL-IgG)  3/90 (anti-β2GP1-IgG) |  |  |  |  |
| Picillo *et al*, 1997 | Italy  *NA* | Unselected patients (1983-1994) | Median duration = 10 (range 1-44) | ELISA (Reaads), ≥ 95^th^ percentile 24 | 27/105 (aCL-IgG/IgM/IgA) |  |  |  |  |
| Pope *et al*, 2000 | Canada  *NA* | Samples from hospital serum data bank | Mean duration = 5.2 years | ELISA (NA), > 15 UI | 3/63 (aCL-IgG/IgM) |  |  |  |  |
| Regéczy *et al*, 2000 | Hungary  *NA* | Random selection of patients (1997-1998) | NA | LA: thromboplastine time  ELISA (homemade), > mean ± 3 SD of controls | 10/43 (aCL-IgG/IgM) |  |  |  |  |
| Renaudineau *et al*, 2001 | Israel  *NA* | Non-selected patients | NA | ELISA (homemade), mean ± 3 SD of controls | 55/478 (aCL-IgG) |  |  |  |  |
| Tektonidou *et al*, 2000 | Greece  *NA* | Consecutive patients | NA | LA: thromboplastine time  ELISA (homemade), > mean ± 3 SD of controls (100 UI) | 0/30 (aCL-IgG/IgM)  0/30 (anti-β2GP1-IgG) |  |  |  |  |
| Touré *et al*, 2013 | Senegal  *NA* | Cross-sectionnal study (2009-2010) | NA | LA: thromboplastine time  ELISA (Diagnostica Stago laboratories), 40 UI/mL | 23/40 (aPL)  2/40 (LA)  7/40 (aCL-IgG/IgM)  20/40 (anti-β2GP1-IgG/IgM) |  |  |  |  |
| Wielosz *et al*, 2009 | Poland  *NA* | Consecutive patients (2005-2007) | 6.2 ± 6.0 yr* | ELISA (Hycor Incorporation) for aCL, 20 UI/mL  ELISA (Euroimmun) for anti-β2GP1, 15 UI/mL | 28/56 (aCL-IgG/IgM+anti-β2GP1-IgG/IgM) |  |  |  |  |

*mean ± standard deviation, NA: not available, ELISA: enzyme-linked immunosorbent assay, LA: lupus anticoagulant, aCL: anti-cardiolipin antibodies, anti-β2GP1: anti-β2GP1 antibodies
